# Supplementary material for: Plastic associated endocrine disruptors reduce Nicastrin protein and potentiate inflammation in hidradenitis suppurativa skin disease
Source: Nat Commun. 2025 Nov 28;16:10755. doi: 10.1038/s41467-025-65789-7 (PMC12663211; doi:10.1038/s41467-025-65789-7)
Supplement: Supplementary file 2 — Supplementary Information [file 41467_2025_65789_MOESM2_ESM.pdf]

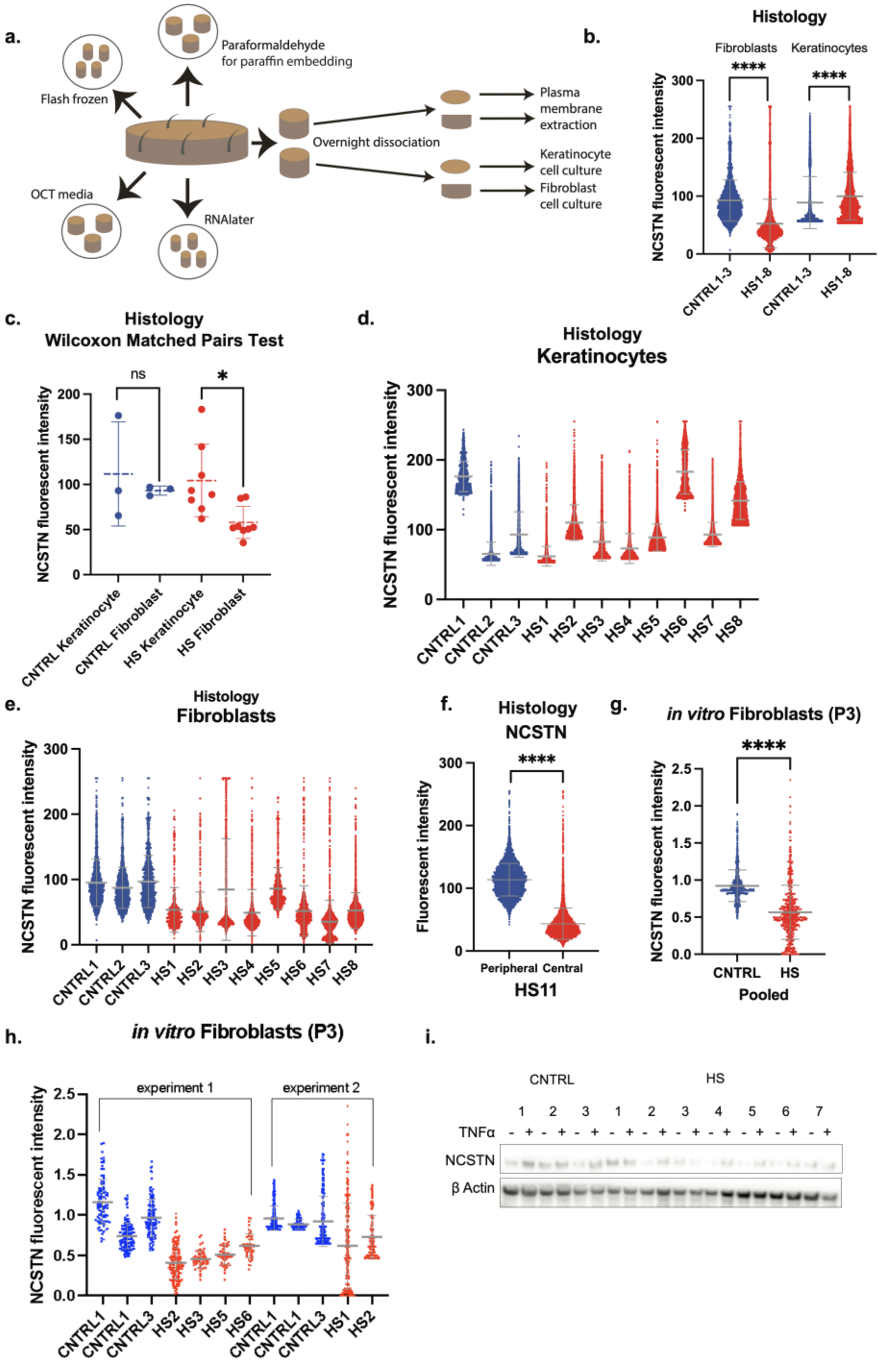

SUPPLEMENTARY FIGURE 1 | Extended patient immunofluorescence data. a, Schematic of patient tissue processing. **b,** NCSTN is low by tissue immunofluorescence in all pooled HS fibroblasts (Welch's t test, $p < 0.0001$) but increased in all pooled HS keratinocytes (Welch's t test, $p < 0.0001$). **c,** There is no difference in NCSTN signal between cell types of CNTRL cells (p = ns), but a significant loss of NCSTN signal in HS fibroblasts compared to keratinocytes in Wilcoxon matched pairs analysis ($p = 0.0156$). **d,** All keratinocytes in CNTRL and HS tissue immunofluorescence for NCSTN (ANOVA $p < 0.0001$). **e,** All fibroblasts in CNTRL and HS tissue immunofluorescence for NCSTN (ANOVA $p < 0.0001$). **f,** NCSTN immunofluorescence signal is higher in the peripheral tissue from an excisional surgery than the central tissue (Welch's t test, $p < 0.0001$). **g,** All pooled *in vitro* HS cells have decreased NCSTN by immunofluorescence compared to *in* vitro CNTRL cells (Welch's t test, $p = 0.0007$). **h,** All fibroblasts in CNTRL and HS *in vitro* immunofluorescence over two experiments (ANOVA, $p < 0.0001$); fluorescent intensities were normalized within each experiment to the CNTRL average. i, Representative western blot of extracted HS and CNTRL fibroblasts with and without TNFα.

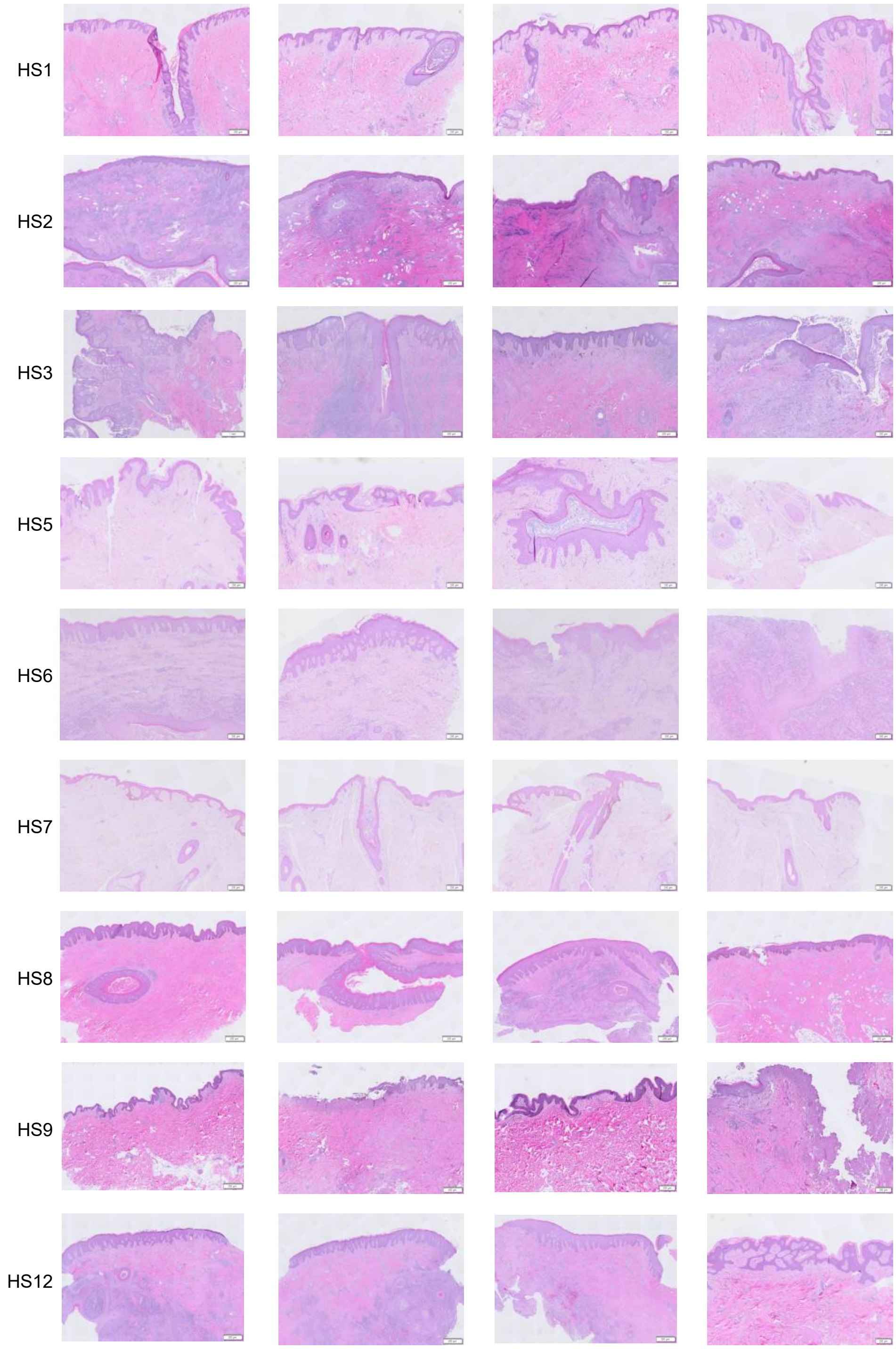

SUPPLEMENTARY FIGURE 2 | H&E images of patient tissue. HS patient identifier on left; 4 representative H&E images are shown for each patient.

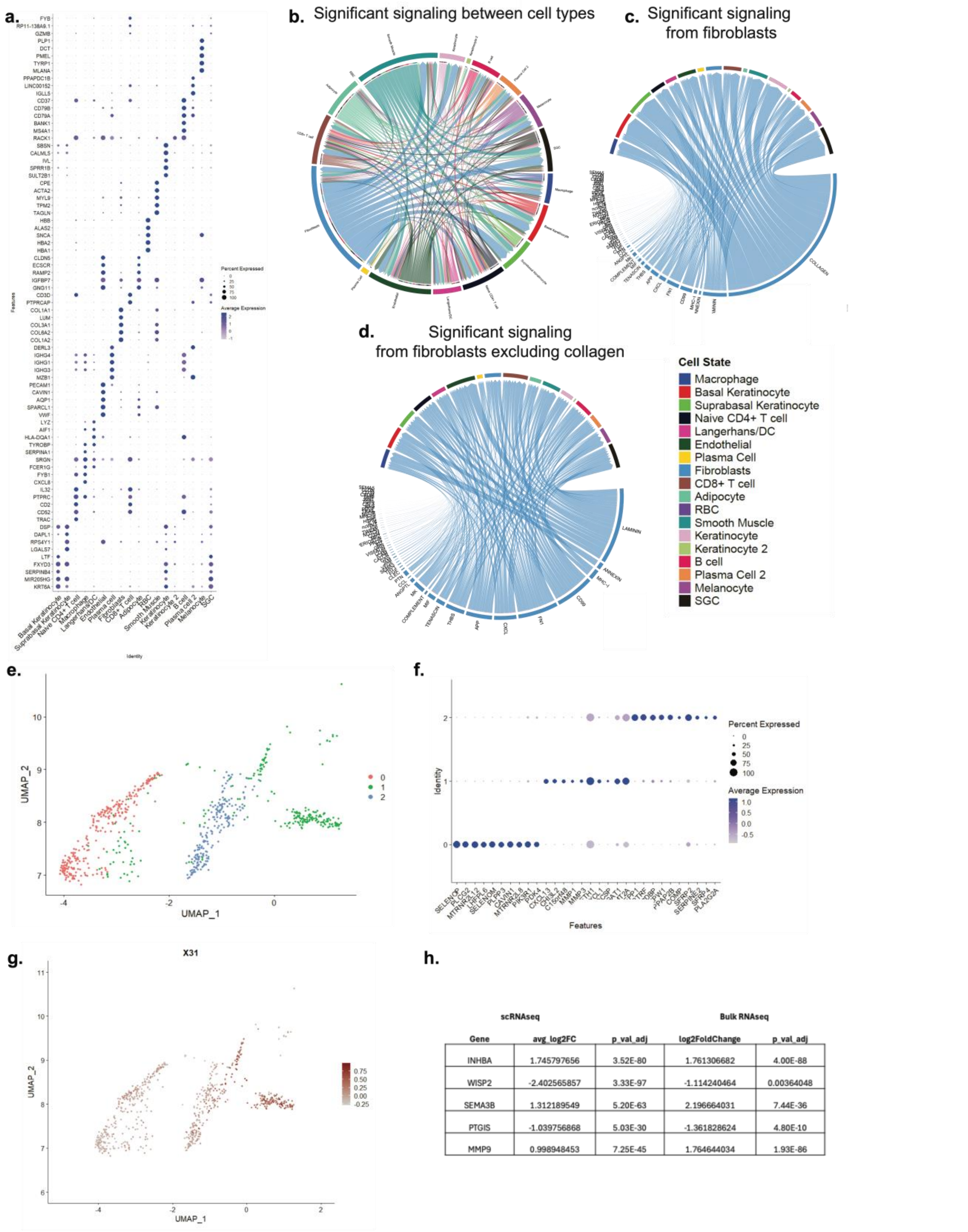

	scRNAseq		Bulk RNAseq	
Gene	avg_log2FC	p_val_adj	log2FoldChange	p_val_adj
INHBA	1.745797656	3.52E-80	1.761306682	4.00E-88
WISP2	-2.402565857	3.33E-97	-1.114240464	0.00364048
SEMA3B	1.312189549	5.20E-63	2.196664031	7.44E-36
PTGIS	-1.039756868	5.03E-30	-1.361828624	4.80E-10
MMP9	0.998948453	7.25E-45	1.764644034	1.93E-86

SUPPLEMENTARY FIGURE 3 | scRNAseq cluster markers and extended signaling. a, Top five markers for each cluster. **b,** Fibroblasts are one of the dominant originators of signaling in a chord diagram of weight of all intercellular signaling between HS cell clusters. **c,** Collagen dominates in a chord diagram of all significant signaling originating from fibroblasts signaling. Removing collagen from the analysis **(d)** highlights more immunology specific signaling pathways. **e,** Sub clustering of the fibroblast cluster reveals three distinct populations with cluster 1 (green) specifically being highly inflammatory and defined by CXCL and MMP gene upregulation (**f**). A modular score for CXCL and CCL genes further highlights fibroblast subcluster 1 as inflammatory (**g**). **h,** cross referencing HS fibroblast scRNAseq and bulk RNAseq of siNCSTN fibroblasts identifies overlaps in differential gene expression.

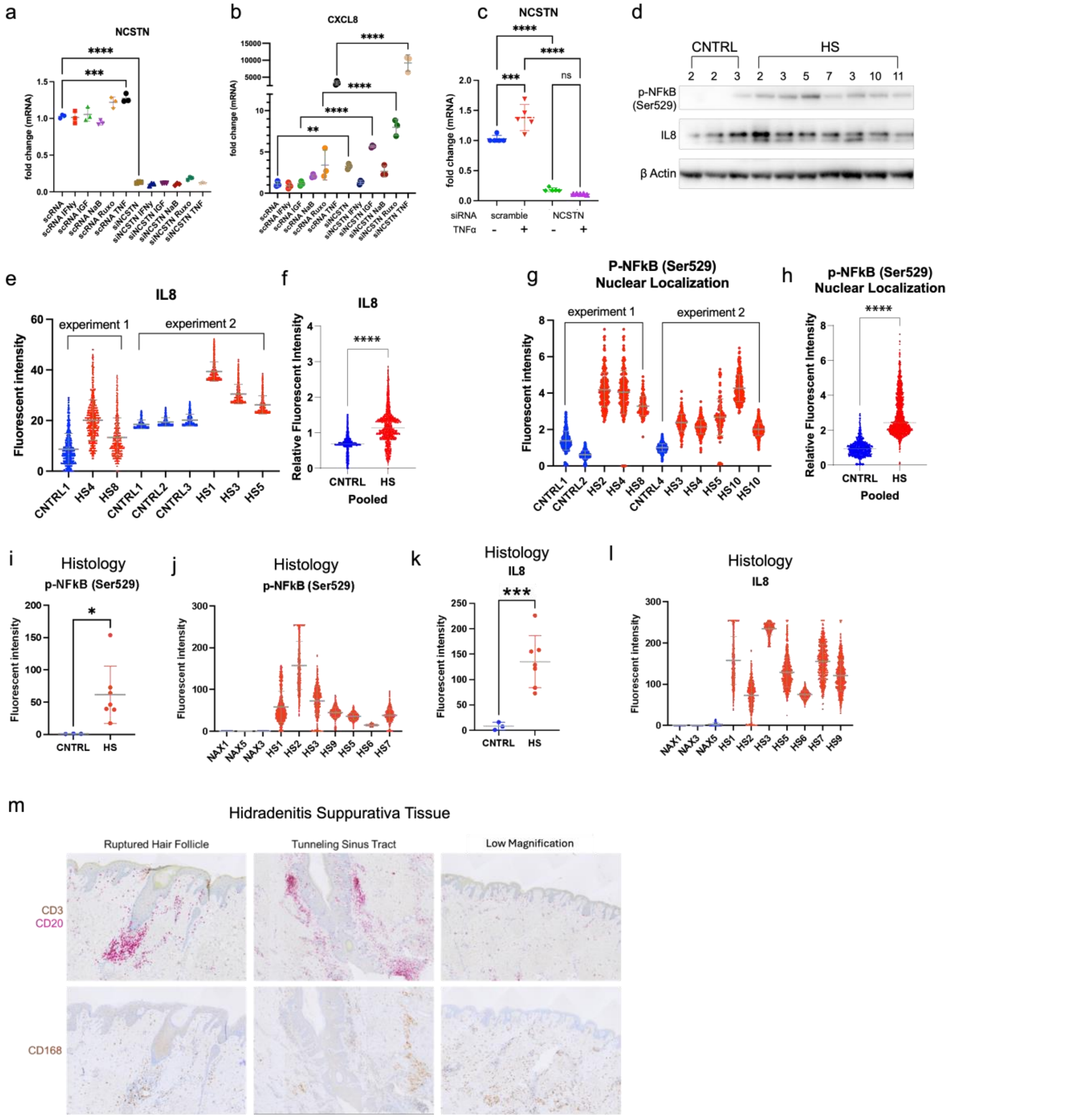

SUPPLEMENTARY FIGURE 4 | Immune screening and extended immunofluorescence data. a, An immune screen performed on normal fibroblasts with control siRNA (scRNA) or siNCSTN was analyzed via RT-qPCR. NCSTN knockdown was validated (ANOVA, $p < 0.0001$) **(a)**, and CXCL8 mRNA was elevated in virtually all siNCSTN immune screen samples compared to scRNA samples. (ANOVA, $p < 0.0001$) **(b)**. **c,** NCSTN knockdown validation for main figure 3a. (ANOVA, $p < 0.0001$) **d,** HS fibroblasts treated *in vitro* with TNFα express more IL8 (Welch's T test, $p = 0.042$) and have higher p-NFκB (Ser529) (Welch's T test, $p = 0.023$) than CNTRL fibroblasts treated with TNFα. **e,** All fibroblasts in CNTRL and HS *in vitro* immunofluorescence for IL8 when treated with TNFα. When these experiments are pooled to compare all CNTRL and all HS fibroblasts, there is significantly more IL8 in HS fibroblasts treated with TNFα ($p < 0.0001$) **(f)**. **g**, All fibroblasts in CNTRL and HS *in vitro* immunofluorescence for nuclear localization of p-NFκB (Ser529) when treated with TNFα. When these experiments are pooled to compare all CNTRL and all HS fibroblasts, there is significantly more IL8 in HS fibroblasts treated with TNFα ($p < 0.0001$) **(h). i,j,** IL8 is significantly higher in dermal fibroblasts of HS tissue than CNTRL tissue immunofluorescence ($p = 0.0167$, n = 3 CNTRL and 7 HS), as is p-NFκB **(k,l)** ($p = 0.0034$, n = 3 CNTRL and 7 HS). **m,** HS tissue has infiltrates of lymphocytes (stained with CD3 and CD20), and neutrophils (stained with CD168) in the dermis.

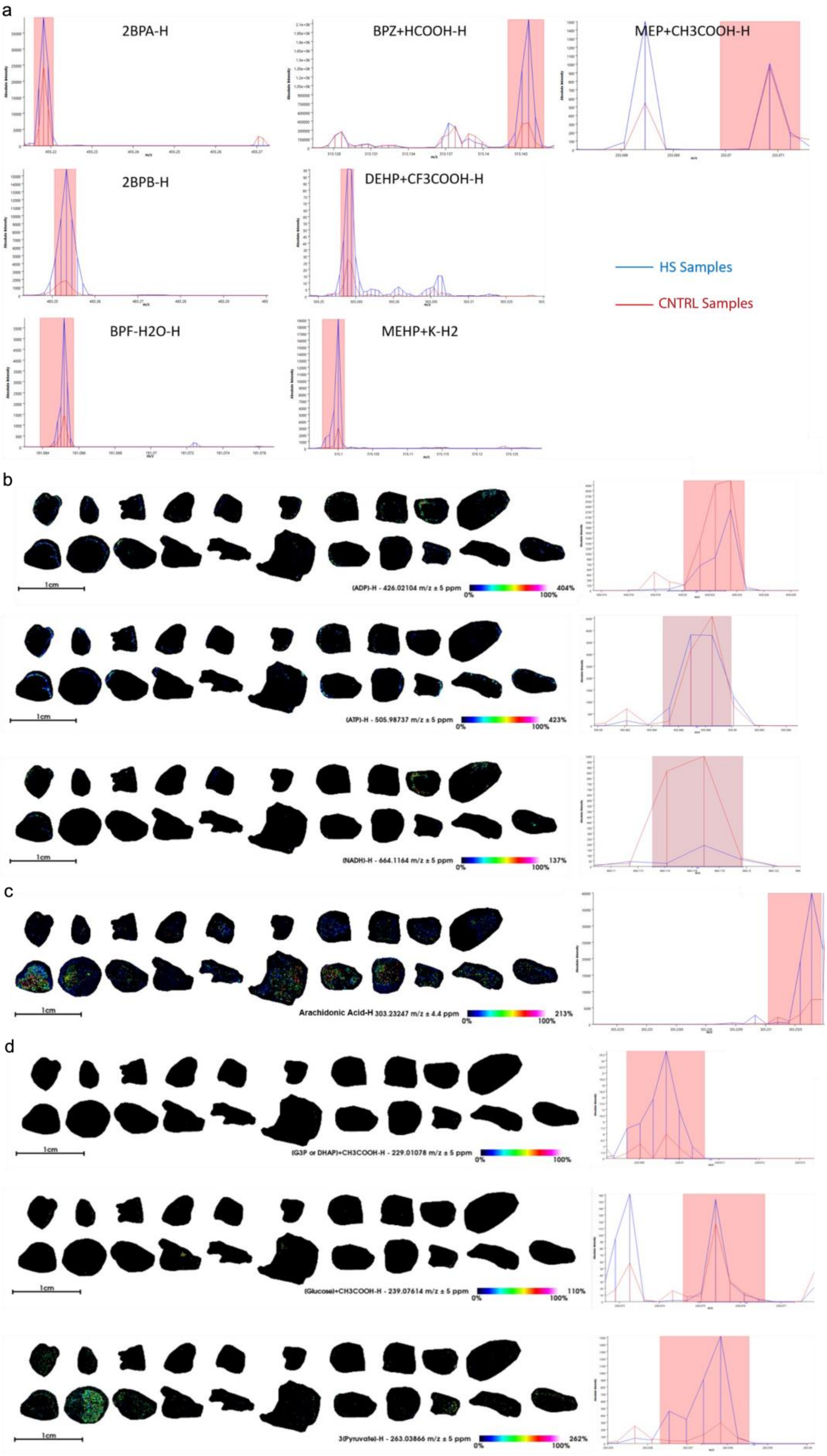

SUPPLEMENTARY FIGURE 5 | Mass spectrometry imaging parent spectra and quality. **a,** The parent spectra of phthalate and bisphenols tested; the overall average of HS spectra (blue lines) is of much greater abundance than the overall average of CNTRL spectra (red lines). **b,** While our MSI protocol was not optimized for detecting ATP and ADP, ADP was observed to be more intense in CNTRL samples when compared to HS samples. In addition, NADH was more intense in CNTRL samples when compared to HS as well. **c,** ATP was detected with similar intensities in both sets of samples. Consistent with HS skin known to have high inflammation, arachidonic acid was detected at much greater abundance than in CNTRL skin. **d,** several glycolytic metabolite candidates were observed despite our protocol not being optimized for them. Parent ions for metabolite candidates G3P or DHAP, glucose, and pyruvate were all observed, with increased intensity for G3P/DHAP and pyruvate in HS samples when compared to CNTRL samples.

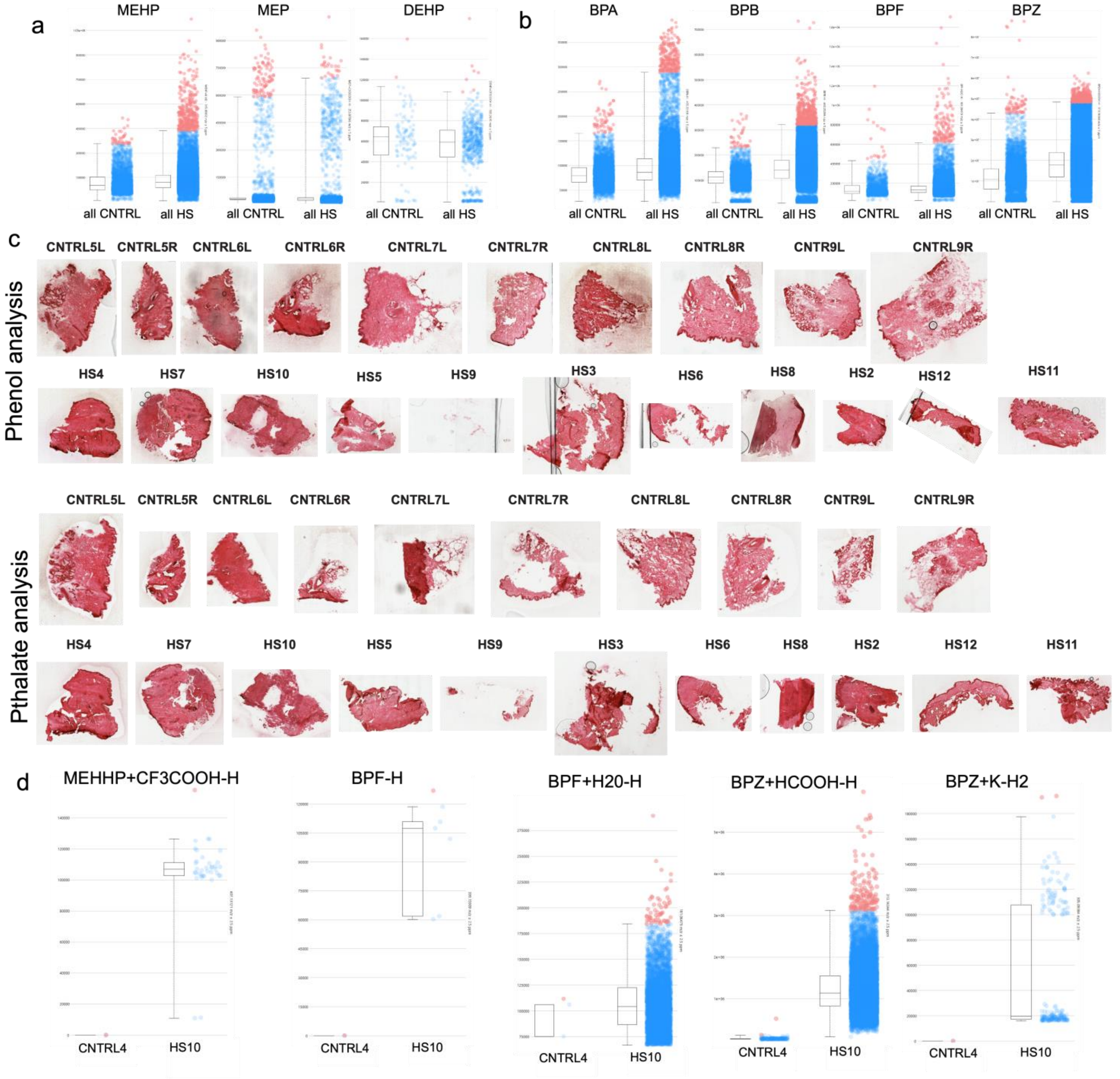

SUPPLEMENTARY FIGURE 6 | Mass spectrometry imaging results. a,b, Quantifications of phthalate (**a**) and bisphenol (**b**) presence in CNTRL vs HS skin on MSI for Figure 4a. All show increased presence in HS skin, with bisphenols having the largest increase between HS and CNTRL. The tissue specimens used for MSI and quantification were H&E stained after analysis (**c**). Quantification was also performed for for MSI analysis of *ex vivo* CNTRL and HS fibroblasts; all showed increased presence in HS fibroblasts (**d**).

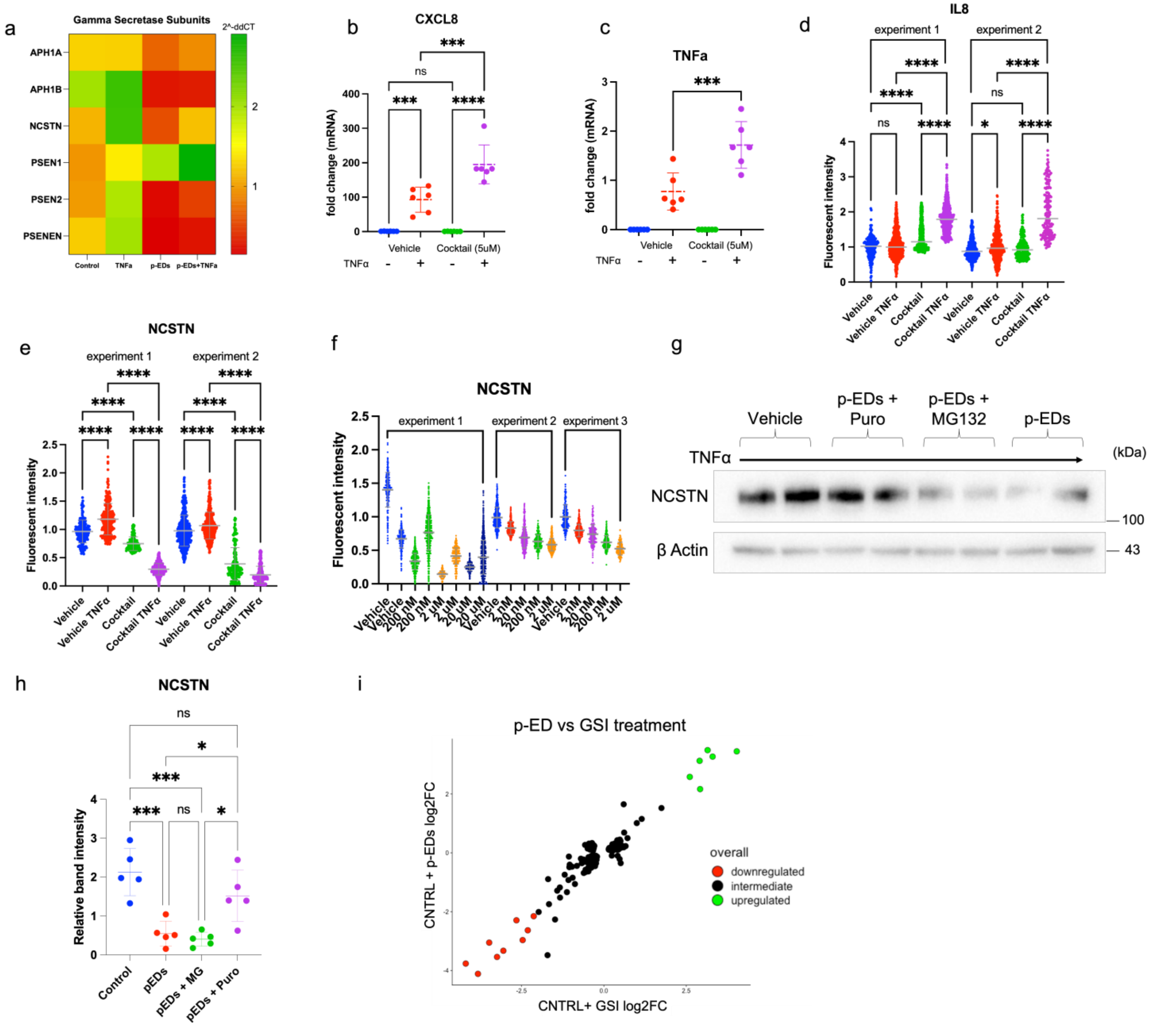

SUPPLEMENTARY FIGURE 7 | Extended p-ED cocktail effect data. a-c, Heat map of RT-qPCR results showing how a 5 uM p-ED cocktail with and without TNFα affects GS members; all GS mRNA is decreased in the presence of p-ED cocktail, with and without TNFα (**a**). Both CXCL8 (**b,** p = 0.0002, n = 6 per condition**)** and TNFα **(c,** p = 0.0001, n = 6 per condition**)** were increased with cocktail TNFα conditions compared to vehicle TNFα conditions. **d-e,** Extended data for experimental repeats on IF for IL8 (**d;** p < 0.0001 overall) and NCSTN (**e**; p < 0.0001 overall). **f,** Extended data for experimental repeats for NCSTN IF cocktail dose response curve. Experiment 2 and 3: NCSTN loss signifcant (p = 0.023 & p = 0.032 respectively) starting at 2 nM. **g-h,** western blot results (representative blot **g**) show puromycin, but not MG-132, can rescue NCSTN loss in p-ED + TNFα treatment conditions (**h**; p < 0.0001 overall, n = 5 per condition) **i,** In a bulk RNAseq experiment, normal axillary fibroblasts treated with gamma secretase inhibitors were transcriptionally nearly identical to normal axillary fibroblasts treated with the p-ED mixture.
